# Posture-Motor and Posture-Ideomotor Dual-Tasking: A Putative Marker of Psychomotor Retardation and Depressive Rumination in Patients With Major Depressive Disorder

**DOI:** 10.3389/fnhum.2018.00108

**Published:** 2018-03-23

**Authors:** Lyubomir I. Aftanas, Olga M. Bazanova, Nataliya V. Novozhilova

**Affiliations:** ^1^Laboratory of Affective, Cognitive and Translational Neuroscience, Institute of Physiology and Basic Medicine, Novosibirsk, Russia; ^2^Department of Neuroscience, Novosibirsk State University, Novosibirsk, Russia

**Keywords:** major depressive disorder, depressive rumination, psychomotor retardation, posture, posture-motor dual-tasking, posture-ideomotor dual-tasking, energy expended for CoPDs

## Abstract

**Background:** Recent studies have demonstrated that the assessment of postural performance may be a potentially reliable and objective marker of the psychomotor retardation (PMR) in the major depressive disorder (MDD). One of the important facets of MDD-related PMR is reflected in disrupted central mechanisms of psychomotor control, heavily influenced by compelling maladaptive depressive rumination. In view of this we designed a research paradigm that included sequential execution of simple single-posture task followed by more challenging divided attention posture tasks, involving concurring motor and ideomotor workloads. Another difficulty dimension assumed executing of all the tasks with eyes open (EO) (easy) and closed (EC) (difficult) conditions. We aimed at investigating the interplay between the severity of MDD, depressive rumination, and efficiency of postural performance.

**Methods:** Compared with 24 age- and body mass index-matched healthy controls (HCs), 26 patients with MDD sequentially executed three experimental tasks: (1) single-posture task of maintaining a quiet stance (ST), (2) actual posture-motor dual task (AMT); and (3) mental/imaginary posture-motor dual task (MMT). All the tasks were performed in the EO and the EC conditions. The primary dependent variable was the amount of kinetic energy (*E*) expended for the center of pressure deviations (CoPDs), whereas the absolute divided attention cost index showed energy cost to the dual-tasking vs. the single-posture task according to the formula: Δ*E* = (*E*_Dual-task_ - *E*_Single-task_).

**Results:** The signs of PMR in the MDD group were objectively indexed by deficient posture control in the EC condition along with overall slowness of fine motor and ideomotor activity. Another important and probably more challenging feature of the findings was that the posture deficit manifested in the ST condition was substantially and significantly attenuated in the MMT and AMT performance dual-tasking activity. A multiple linear regression analysis evidenced further that the dual-tasking energy cost (i.e., Δ*E*) significantly predicted clinical scores of severity of MDD and depressive rumination.

**Conclusion:** The findings allow to suggest that execution of concurrent actual or imaginary fine motor task with closed visual input deallocates attentional resources from compelling maladaptive depressive rumination thereby attenuating severity of absolute dual-tasking energy costs for balance maintenance in patients with MDD.

**Significance:** Quantitative assessment of PMR through measures of the postural performance in dual-tasking may be useful to capture the negative impact of past depressive episodes, optimize the personalized treatment selection, and improve the understanding of the pathophysiological mechanisms underlying MDD.

## Introduction

Psychomotor retardation (PMR) reflects one of the central dimensions of major depressive disorder (MDD). Most descriptions of PMR emphasized disturbances in speech; facial expression; fine motor behavior; gross locomotor activity; or ideation along with respective EEG, PET, and fMRI concomitants (Bennabi et al., 2013; Cullen et al., 2015; Liberg and Rahm, 2015). In more general concept, the symptoms and signs of the PMR, therefore, entail a wide range of brain functions executive function, emotion, volition, and drive (Liberg and Rahm, 2015). Correlational analyses between signs of PMR and severity of depression indicate that some aspects of psychomotor slowing are state-dependent, whereas some others might be trait-dependent and this issue still remains to be clarified (Bennabi et al., 2013). Combination of different experimental techniques for measuring PMR with conventional clinical assessments by subjective observer-rated scales or by single items in specific depression rating scales could offer an increased understanding of PMR in depression. In this respect, a more quantitative assessment of PMR through objective measures may be useful to capture the negative impact of past depressive episodes, optimize the personalized treatment selection, and improve the understanding of the pathophysiological mechanisms underlying different depressive subtypes of depression (Bennabi et al., 2013; Wallace et al., 2013; Liberg and Rahm, 2015; Thomas-Ollivier et al., 2017).

Although few, there are findings (Deschamps et al., 2014, 2016) that among objective measures, a center of pressure deviations (CoPD) velocity-based posture control assessment could be a reliable objective marker of PMR in MDD patients.

Several studies showed greater posture instability in EC condition in patients with MDD compared to healthy control (HC), which was likely related to deficits in the integration of visual and proprioceptive inputs necessary for efficient posture control (Deschamps et al., 2014, 2015).

Studies of another type investigated the disruptive effects of depression on seemingly automatic by nature postural performance in divided attention tasks (Doumas et al., 2012; Deschamps et al., 2015). In terms of posture control assessment, the absolute dual-tasking cost for a given task is simply the amount by which mean of the path of CoPDs increased from single- to dual-task conditions. The researchers investigated dual-tasking costs by combining low or highly challenging cognitive tasks with primary posture control (Doumas et al., 2012; Deschamps et al., 2015; Bazanova et al., 2017). Findings from these dual-tasking investigations posture–cognitive paradigms evidenced executive control deficits in MDD patients that affect both cognitive and sensorimotor task performance (Doumas et al., 2012; Deschamps et al., 2015). The absolute costs in dual posture–cognitive tasks significantly correlated with PMR-specific clinical scales (Deschamps et al., 2016) and reliably documented the efficiency of walking exercise and TMS-treatment programs recommended for people with MDD (Deschamps et al., 2015, 2016).

In order to expand this still scarce body of evidence attesting highly promising discriminative properties of the CoPD assessment as a depression-related correlate of the PMR, we designed a research paradigm that included sequential execution of a single-posture and divided attention posture tasks in eyes open (EO) and closed (EC) conditions. Unlike other investigations, our dual-tasking procedure assumed the sequential performance of the same fine motor task, executed in the actual and mental/imaginary versions. Such an approach allowed us to separate motor and ideomotor components of concurrent activity within the same divided attention paradigm.

The investigation reported in this article was carried out with three main issues in mind.

(1) The first one was to examine whether dynamics of the energy (*E*) expended for balance maintenance in the newly designed paradigm discriminates HC and patients with MDD. For the single task, we anticipated impaired posture control as indexed by increased *E* demands for balance maintenance in the EC condition for both groups, with significantly more disruptive impact in patients in the single task. That could be mainly due to their depression-related deficit in integration of visual and proprioceptive inputs (Bolbecker et al., 2001; Doumas et al., 2012; Deschamps et al., 2016).
(2) For the divided attention performance, our prediction was more sophisticated. It has been documented that depressive rumination consumes large amounts of attentional resources accounting for depression-linked PMR signs of overall psychomotor slowness (see e.g., Nolen-Hoeksema, 2000; Hamilton et al., 2011). We suggested that execution of the actual or even imaginary concurrent motor task would deallocate attentional resource from compelling depressive rumination, thereby attenuating the amount of dual-tasking absolute *E* costs in patients with MDD.
(3) The third idea was to estimate predictive value of absolute *E* dual-tasking costs with respect to clinical scores of severity of depression and rumination.

## Materials and Methods

### Participants

Fifty right-handed participants (the age range from 20 to 55 years old) were recruited to the study. Patients (*n* = 26) were selected from Institute’s Outpatient Psychotherapy Clinic («A1 Clinic») and screened by one of three psychiatrists (YL, A.M., and L.A.) using the Structured Clinical Interview for DSM-V – (SCID, First et al., 2016) to identify cases of MDD. Those patients who were diagnosed with the MDD were included in the MDD group (American Psychiatric Association, 2013). The control (non-depressed) subjects (*n* = 24) were recruited following advertisement. All the participants were required to refrain from taking any psychotropic medication at the time of the study and were free from alcohol at least 3 weeks before. All the participants were interviewed with the Hamilton Rating Scale (HDRS-17, Hamilton, 1960), and completed the Beck Depression Inventory-II (BDI-II, Beck et al., 1996) and the Ruminative Responses Scale (RRS, Nolen-Hoeksema, 2000). All study participants provided written informed consent and the study was approved by the Ethical Committee of State Research Institute of Physiology and Basic Medicine (Novosibirsk, Russia).

### Apparatus and Procedure

Stabilometric recordings of CoPD were performed using the force-platform system linked to ST-150 software (sampling frequency of 33 Hz) (BioMera, Moscow, Russia).

### Actual Fine Motor and Ideomotor Dual Tasks

Fine motor control is associated with tasks that typically involve some form of accurate manual manipulation. We administered actual-timed finger motor tasks (AMTs) in the form of sequential finger tapping – opposing each finger with the thumb in sequence, i.e., “index, middle, ring, little” comprises a set. Subjects reported the number of sets completed after the performance (Cambridge-Keeling, 2002). Before the test, the examiner gave verbal instruction while demonstrating the expected performance to ensure that the participant understood the instruction. Brief, untimed practice followed. After practicing the examiner said, “When I say ‘go’, do the same thing as fast as you can until I stop you.” The fluency of fine motor task performance was calculated by dividing the number of sets by the total amount of performance time as a number of sets per minute (set/min). In the mental motor task (MMT) condition, participants performed the same task, but instead of actually moving their fingers they were asked to imagine performing the movements. Participants were explicitly and repeatedly instructed to maintain the vividness of motor imagery throughout the experiment. All participants also calculated a number of sets to ensure that they were able to engage imagery and would be able to perform the task according to the instructions. Both actual and mental imaged motor tasks were performed in standing position.

### Procedure

The energy expended for CoPD was performed using three experimental tasks: (1) single-posture task as a quiet stance (ST), (2) actual dual-posture fine motor task (AMT); and (3) dual-posture mental/imagery version of the fine motor task (MMT). The EO and EC conditions were counterbalanced within the groups with the restriction that MMT always followed AMT. In the EO condition, the subjects looked at a fixed level target at a distance of 2 m. The participants were instructed to maintain an erect position for 30 s under each condition, with arms alongside the body and feet positioned on a schematic representing a 30° angle with respect to the anteroposterior direction. Two trials (each of 30 s duration) were performed for each condition. The results were averaged across trials. Those trials where artifact movement of eyes, head, arms or legs were video-recorded with webcam were excluded from the analysis.

### Dependent Variables

For a trial of 30-s duration (sampling frequency of 33 Hz), the force-platform system was linked to ST-150 software (Biomera, Moscow, Russia), thus providing CoP series on the anteroposterior (Y) and mediolateral (X) axes assessment (Grohovsky and Kubryak, 2014). The dependent variables were calculated from the analysis of CoP trajectories. Trace length (L) in millimeters of CoP excursion and sway velocity (V in m/s) corresponding to the sum of the CoPD scalars divided by the sampling time were normalized to the participant’s height (Rocchi et al., 2004; Bazanova et al., 2017). The amount of kinetic energy (*E*) expanded for balance maintenance (mJ/kg) was calculated according to the formula

$$\begin{array}{c} E=\sum_{i\;=\;1}^{n}m^{*}(V_{i+1}^{2}-V_{i}^{2})/2 \end{array}$$

where *m* is the body weight, *V* the CoPD velocity, and *n* the number of discrete measurements conducted (Grohovsky and Kubryak, 2014).

To quantify subjects’ ability for executing two tasks concurrently, we calculated for each subject and task the absolute dual-task costs (McDowd, 1986), namely, the absolute dual-task energy costs index (i.e., Δ*E*), showing individual cost to the dual-tasking versus the single-posture task according to the formula: Δ*E* = (*E*_Dual-task_ - *E*_Single-task_).

### Statistical Analysis

IBM SPSS statistic software was used to analyze data. Normality was ensured by Kolmogorov–Smirnov test. A three-way ANOVA with the factors of Group as between (Group 2: HC vs. MDD), and Vision (Vision 2: EC vs. EO) and Task (Task 3: ST vs. dual AMT vs. dual MMT) as repeated measures were performed and were followed by separate ANOVAs and planned comparisons. The Bonferroni correction was applied where appropriate.

In line with our primary purpose, correlation analyses were performed to assess interplays among clinical, posture, and motor fluency variables. A forward stepwise multiple regression analysis was used to determine if posture assessment variables under dual-tasking paradigms would predict clinical scores of MDD and compelling depressive rumination.

## Results

### Group Characteristics

As shown in **Table 1**, participants in the depressed group reported significantly greater symptoms of depression and depressive rumination than those in the control group: HDRS-17, *t*(48) = 18.127, *p* < 0.001; BDI-II, *t*(47) = 13.92, *p* < 0.001; RRS, *t*(47) = 8.35, *p* < 0.001. Groups did not differ in age, *t(*48) = 0.099, *p* > 0.921, gender ratio, *X*^2^ (1, *n* = 48) = 1.864, *p* > 0.397, education level, *X*^2^ (1, *n* = 50) = 0.162, *p* > 0.685, or marital status, *X*^2^ (2, *n* = 50) = 0.541, *p* = >0.760.

**Table 1:** Differences in demographic and clinical characteristics between groups.

Group	HC	MDD
Age, *M*(1 *SD*)	36.21 (6.6)	36.23 (9.6)
*n*	24	26
Education level (%)		
High School	13.09	20.0
University	86.1	80.0
Marital status (%)		
Married	83.3	57.5
Single	16.07	42.5
Separated/divorced	0.00	0.00
HDRS-17, *M*(1 *SD*)	2.52 (2.81)	16.76 (2.68)
BDI-II, *M*(1 *SD*)	3.71 (2.97)	33.44 (10.14)
RRS, *M*(1 *SD*)	35.86 (10.97)	60.21 (10.01)

#### AMT and MMT Fluency Data

Individual scores of the motor fluency in dual-tasking paradigms were subjected to a three-way ANOVA [2 (Group: HC vs. MDD) × 2 (Vision: EO vs. EC) × 2 (Task: AMT vs. MMT)]. The analysis revealed: (1) the significant main effect of Group [F(1,46) = 28.08, p < 0.001] indexed overall better performance in HC vs. MDD patients; (2) the main effect of Vision [F(1,48) = 6.482; p = 0.013] showing faster performance for both actual and ideomotor tasks in the EO vs. EC condition; and (3) the main effect of Task [F(1, 48) = 96.826, p = 0.001] exhibited overall better fluency for the AMT vs. MMT, manifesting slower performance for the latter one.

Analysis of the specific three-way interaction involving the factor of Group [Group × Vision × Task: *F*(1,46) = 6.730, *p* = 0.013] revealed that patients, but not controls exhibited poorer fluency for the AMT in EC vs. EO conditions (*t* = -6.87, *p* < 0.001). By contrast, the HC group acted equally well in both the tasks regardless of the presence or lack of visual information (**Table 2**).

**Table 2:** Actual motor task (AMT) and imaginary motor task (MMT) fluency scores (mean, 1 *SD*) of healthy control (HC) and MDD groups in dual-tasking paradigm.

group		HC		MDD	
*n*		24		26	
		EO	EC	EO	EC
AMT fluency (sets/min)	*M*	28.71	29.33	22.62ˆ	20.00ˆ
	*SD*	5.66	5.57	5.15	4.83
MMT fluency (sets/min)	*M*	24.42	21.58^∗^	15.58^∗^ˆ	15.92^∗^ˆ
	*SD*	7.68	6.57	3.88	4.62

*^∗^*p* < 0.001, AMT vs. MMT; ˆ*p* < 0.001, HC vs. MDD.*

#### Kinetic Energy Expanded for Balance Maintenance (E) as a Dependent Variable

**FIGURE 1:**
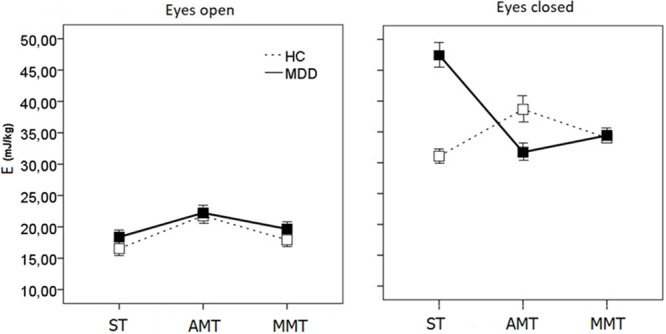
Energy demands for CoPD (*E*) scores during single- (ST), dual-motor (AMT), and dual-mental/ideomotor (MMT) tasks in EO and EC condition in HC and patients with MDD. Error bars represent standard errors.

A three-way ANOVA [2 (Group: HC vs. MDD) × 2 (Vision: EO vs. EC) × 3 (Task: ST vs. AMT vs. MMT)] was performed for the dependent variable of *E* and it revealed a significant main effect of Vision [*F*(1,46) = 90.977, *p* = 0.000)]. The Vision effect highlighted larger energy expenses for EC vs. EO conditions in both groups, whereas the three-way interaction Group × Vision × Task [*F*(2,278) = 18.76, *p* < 0.001] showed that between-group differences depend on both factors of Vision and Task (**Figure 1**). Inspection of the means of this interaction followed by separate ANOVAs for each visual condition [Group (2) × Task (3)] points to the EC one as the factor creating a crucial context for discriminating groups (**Figure 1**).

In the EO conditions, one can see some simple tendency of HC to perform with a little bit lesser energy expenses than MDD in all the three tasks {Group [*F*(2,46) = 1.342, *p* < 0.052], **Figure 1**}. Much more complicated picture of intergroup differences emerges in the EC condition {Group × Task [*F*(2,47) = 20.612, *p* < 0.001]}.

On the one hand, HCs are energetically less demanding than patients {Group [*F*(2,47) = 13.672, *p* < 0.001]}. Inspection of the means of the Group × Task {[*F*(2,47) = 9.712, *p* < 0.002]} interaction evidences that both groups exhibited different patterns of energy demands changes while performing single and dual tasks. Namely, the ST is energetically much more demanding for the patients with MDD than HC (planned comparison *t* = 7.97 at *p* < 0.001), while the AMT and MMT dual-tasking paradoxically drops the energy consumption down for MDD (AMT, *t* = -7.949, *p* < 0.001; MMT, *t* = -3.369, *p* < 0.003). And vice versa, dual-tasking increases energy demands values in controls (AMT, *t* = 6.287, *p* < 0.001; MMT, *t* = 3.199, *p* < 0.004) leveling them up to those of MDD patients (**Figure 1**).

#### Absolute Dual-Task Energy Costs Index (Δ*E*)

**FIGURE 2:**
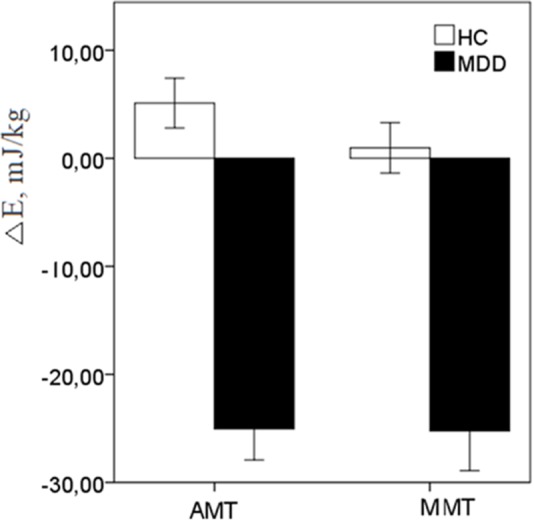
The energy reactivity index (Δ*E*) in response to dual-motor (AMT) and dual-mental/ideomotor (MMT) tasks in the EC condition in HC and patients with MDD. Error bars represent standard errors.

A two-way ANOVA [2 (Group: HC vs. MDD) × 2 (Task: AMT vs. MMT) of the Δ*E* (*E*_Dual-task_ - *E*_Single-task_)] scores revealed robust effect of Group {[*F*(1,48) = 49.08, *p* < 0.0001)} thereby reliably documenting that in the EC condition under posture-actual-motor (*F* = 43.93, *p* < 0.001) and posture-mental-motor dual-tasking (*F* = 18.39, *p* < 0.001) HC increased, while patients with MDD reduced energy demands (**Figure 2**).

Since the fluency rates under the dual-tasking paradigm significantly differed between the groups, and positively associated with Δ*E* scores in AMT (*r* = 0.654, *p* = 0.002) and in MMT (*r* = 0.612, *p* = 0.004) in EC condition, the ANOVAs of the Δ*E* scores in EC were followed by univariate ANCOVAs [2 (Group: HC vs. MDD)] with respective fluency covariates. Even though fluency covariates substantially degraded the main effects of the factor of Group for dual-tasking energy costs, the corrected effects still remained robust and highly significant [AMT: *F*(1,49) = 10.315, *p* < 0.002; MMT: *F*(1,49) = 9.522, *p* < 0.003].

### Correlational Analyses

Correlational analyses (Pearson correlation) for the whole sample showed that fluency scores for the AMT and MMT tasks in both visual conditions negatively correlated with the HDRS-17, BDI-II, and RRS scores. Meanwhile, the *E* variables positively correlated with HDRS-17, BDI-II, and RRS score only in the single-task EC condition (*r* = -0.567, *p* < 0.004). On the other hand, the Δ*E* in AMT, Δ*E* in MMT both negatively associated with HDRS-17, BDI-II, and RRS scores (**Table 3**). The findings from regression analysis revealed that the Δ*E* in AMT and Δ*E* in MMT significantly predicted clinically relevant scores of depression and compelling depressive rumination (**Table 4**).

**Table 3:** Bivariate correlation of fluency scores, depressive, and ruminative symptoms.

		EO			EC		
		Depressive symptoms			Depressive symptoms		
		BDI	HDRS	RRS	BDI	HDRS	RRS
AMT fluency (set/min)	*r*	–0.520^∗^	–0.577^∗^	–0.507^∗^	–0.630^∗^	–0.694^∗^	–0.619^∗^
	Sig	0.001	0.001	0.001	0.001	0.001	0.001
	*n*	49	49	47	49	49	47
MMT fluency (set/min)	*r*	–0.335	–0.515^∗^	–0.362^∗^	–0.292	–0.490^∗^	–0.334
	Sig	0.022	0.002	0.013	0.047	0.004	0.023
	*n*	47	47	46	47	47	46

*^∗^, significant differences at *p* < 0.007; HDRS-17, Hamilton Depression Rating Scale; RRS, Ruminative Response Scale.*

**Table 4:** Multiple stepwise linear regression analysis of the *E* and Δ*E* in the single-posture task (ST), dual-posture actual motor (AMT), and dual-posture mental/ideomotor (MMT) tasks as independent predictors of depressive and ruminative symptoms (HDRS-17, BDI, and RRS scores).

Dependent variables	Steps	Predictors	Beta	*r*_partial_	*r*_semipartial_	Adjusted *r*	*F*	*p*
HDRS-17 score	1	Δ*E* in AMT	–0.875	–0.875	–0.875	0.761	147.423	0.001
	2	Δ*E* in AMT	–0.147	–0.714	–0.433	0.812	100.401	0.001
		Δ*E* in MMT	–0.212	–0.481	–0.233			0.001
BDI-II score	1	Δ*E* in AMT	–0.872	–0.872	–0.872	0.756	143.240	0.001
	2	Δ*E* in AMT	–0.617	–0.683	–0.406	0.802	94.441	0.001
		Δ*E* in MMT	–0.340	–0.458	–0.224			0.001
	3	Δ*E* in AMT	–1.0065	–0.701	–0.387	0.834	78.162	0.001
		Δ*E* in MMT	–0.3316	–0.485	–0.218			0.001
		*E* in ST/EC	0.426	0.424	0.184			0.004
RRS score	1	Δ*E* in MMT	–0.698	–0.698	–0.405	0.578	22.089	0.001
		Δ*E* in AMT	–0.489	–0.532	–0.309			0.001

*E in ST/EC = energy expended for balance maintenance in single-posture task in EC.*

## Discussion

As we expected, the signs of PMR in the MDD group were objectively indexed by posture control assessment scores and by fluency performance data in both the AMT and MMT tasks showing deficient posture control in the EC and not in the EO condition along with overall slowness of fine motor and ideomotor activity. Another important and probably more challenging feature of the findings was that posture deficit of patients with MDD manifested in the single-task condition was substantially and significantly attenuated by the MMT and AMT dual-tasking activity. A multiple linear regression analysis evidenced further that the dual-tasking energy cost significantly predicted clinical scores of severity of MDD and depressive rumination.

### Energy Costs for Postural Performance Under Visual and Nonvisual Condition

The rationale of manipulation with the visual input was that the EC condition makes the visual information unavailable, and therefore proprioceptive inputs must be predominantly used for an effective “automatic” posture control. When associated with the impaired integration of proprioceptive inputs in MDD, the essential “proprioceptive reweighting” process (Peterka and Loughlin, 2004) for maintaining posture control would be adversely influenced by divided attention performance (Barr et al., 2016; Deschamps et al., 2016). It is well documented that posture control abilities in humans depend on the capacities to detect the environment and visual, vestibular, and somatosensory inputs (see for review Newton, 1997; Forbes et al., 2015). More specifically, this is achieved primarily by a feedback-control mechanism that includes dynamic regulation of sensorimotor integration by the “reweighting of individual sensory channels” (Peterka and Loughlin, 2004). Vision and visual and spatial attention are among executive functions that are essential for successful posture stability and navigation through the environment (e.g., Albert et al., 1999; Nagamatsu et al., 2013). EC condition makes the visual information unreliable, and therefore remaining sensory inputs must be predominantly used for an effective “automatic” posture adjustment while a “load-compensation” mechanism attempts to modulate the magnitude of corrective torque needed to achieve good control of ST (Peterka and Loughlin, 2004; Bolbecker et al., 2011). As it was predicted, there was the significantly heavier impact of closed visual input in patients vs. HCs. Poorer posture stability in patients suggested an inability to compensate decreasing of sensorial inputs (vision), which could be mainly due to the PMR-related deficit of sensorimotor integration in depression (Bolbecker et al., 2001; Doumas et al., 2012; Deschamps et al., 2016). If so, it is associated with deficits in the integration of vestibular and proprioceptive inputs, the essential “reweighting of individual sensory channels” led to deficient posture control (Deschamps et al. 2015, 2016).

### Fluency in Fine Motor and Ideomotor Tasks

Before going to the dual-tasking and *E* costs for posture control, let us first have a closer look at the fluency data. Our main findings on these revealed a global slowness of both actual and mental movements in patients. Another distinctive feature was that fluency of imagined movements, as those of actual movement, was poorer in depressed patients, whereas controls acted equally well in both tasks. Our observation corresponds with previously described psychomotor deficits in unipolar depression (Bennabi et al., 2013, 2014; Liberg and Rahm, 2015; Thomas-Ollivier et al., 2017) and could be related to functional and structural changes within specialized brain circuits, associated with negative affect, cognitive and motor control, and action representation (Schrijvers et al., 2008; Walther et al., 2012; Liberg et al., 2013).

### Energy Costs in the Single vs. Dual Tasks

In line with our prediction, during execution of the actual or even imaginary concurrent motor task patients manifested reliable and statistically significant attenuation of energy expended for balance maintenance.

In an attempt to outline the possible mechanisms for such an improvement, we had accepted the following logic of reasoning.

On the one hand, according to the capacity model of attention (e.g., Kahneman, 1973), there is a finite reserve of cognitive resources, i.e., the more resources the given task requires, the fewer functional space that might be distributed to a secondary task is available. Dual-tasking cost could be a valid marker of frailty and a useful tool for identifying persons with signs of PMR (Lundin-Olsson et al., 1998). Simultaneous performance of an attention-demanding task (cognitive or psychomotor) during posture/gait tasks often leads to profound negative effects not only on gait and balance but also on the efficiency of cognitive task performance (Woollacott and Shumway-Cook, 2002; Al-Yahya et al., 2011). Such reductions in performance have been commonly interpreted as competition for attentional resources between the posture and cognitive task.

On the other hand, ruminative responding in MDD is defined as a recurrent, self-reflective, and unintentional focus on depressive symptomatology and its causes and consequences. A trait-like ruminative response style has been found to predict higher levels of depressive symptoms in depressed individuals, perhaps because of disrupted allocation of cognitive resources and increased recall and rehearsal of negative life events (Nolan et al., 1998; Kuehner and Weber, 1999; Nolen-Hoeksema, 2000). The depressive rumination overcrowds attentional resources, thereby accounting for the PMR signs of cognitive slowness and deficient psychomotor activity in MDD patients (Nolen-Hoeksema, 2000; Hamilton et al., 2011; Machino et al., 2014). Recent studies showed that of the two distinct and anti-correlating neuronal networks, i.e., the default-mode network (DMN) and the task-positive network (TPN), the first one may represent important neural substrates of depressive rumination (e.g., Hamilton et al., 2011). Performance of attention-demanding tasks activates the TPN circuits and reciprocally suppresses the DMN activity. During wakeful rest, the opposite pattern emerges, when the activated DMN lead to outbursts of depressive rumination in patients with MDD (Hamilton et al., 2011).

Let us put up together all of the above. Hypothetically, execution of the single-posture task in the EC condition increases the relative dominance of the DMN that propels depressive rumination accompanied by consumption of additional attentional resources. The dual-tasking “switches off” the DMN activity with inherent depressive rumination and redirect released attentional resource toward the secondary tasks and adaptive engagement of the TPN. As a result, rumination-linked posture deficit in patients with MDD is attenuated (Nolen-Hoeksema, 2000; Hamilton et al., 2011; Machino et al., 2014). Executing fine motor and ideomotor task performance might have an immediate positive effect on the limbic system, lead to changes in functionally connected remote areas, enhance activity of the hypo-activated regions and pathways, strengthen weakened frontostriatal coupling, and finally improve sensor-motor integration (Lohr et al., 2013; Baune et al., 2014; Roca et al., 2015).

## Conclusion

Overall, the findings from the CoPD assessment variables allow us to suggest that execution of concurrent motor actual or imaginary finger movement task with closed visual input deallocates attentional resource from compelling maladaptive depressive rumination (such as e.g., brooding) thereby improving the depression-related PMR. The findings expand previously reported evidence (Doumas et al., 2012; Deschamps et al., 2014, 2015, 2016) that the assessment of posture performance could be a reliable marker of PMR in patients with MDD to the younger population. We are also the first to evidence significant association of deficient posture performance with depressive rumination in patients with MDD.

Some limitations of the study need to be considered. Our results must be viewed with caution as they need further investigation and generalization. The effect of severity and state of depression needs to be analyzed in longitudinal studies, including neuropsychiatric control groups and depressive subgroups. More sophisticated metrics and control instruments (video-control, VAS scales for monitoring motivational factors including interest, pleasure, willingness, and effort) of the actual motor and ideomotor tasks, being executed, should be applied.

The findings from posture-based dual-tasking paradigm suggest the development of a putative posture-based dual-tasking biofeedback training technology, aimed to deallocate attentional resource from compelling depressive rumination thereby attenuating the depression-related PMR signs of deficient sensorimotor integration (see e.g., Giggins et al., 2013). Combination of different experimental techniques for measuring postural disturbance with clinical assessments could offer an increased understanding of MDD-related PMR, better prediction of individual response to therapy, or depression risks.

## Ethics Statement

The study has been approved by the Institute’s local Ethical Committee in accordance with the Declaration of Helsinki. Trained interviewers administered the Structural Clinical Interview for the DSM-V (SCID, First et al., 2016) to patients during their first session in the study and those with primary diagnosis of MDD were included in the depressed group. Individuals with current or lifetime diagnosis of bipolar disorder, psychotic symptoms, and those who abused alcohol or other substances within the past 6 months were excluded. The HC group consisted of individuals who did not have a current diagnosis or past history of any Axis I disorder according to the SCID. All participants were between the age of 20 and 52 years and normal or corrected-to-normal vision. All individuals were required to refrain from taking any psychotropic medication at the time of the study. All the participants were interviewed with the Hamilton Rating Scale (HDRS-17, Hamilton, 1960) and completed the BDI-II (Beck et al., 1996) and the RRS (Nolen-Hoeksema, 2000).

## Author Contributions

LA had leading contributions to the main concept of the work, analysis, and interpretation of data, revising it critically for important intellectual content, and final approval of the version to be published. OB had substantial contributions to the design, acquisition, analysis, interpretation of data, revising it critically for important intellectual content and final approval of the version to be published. She provided agreement to be accountable for all aspects of the work in ensuring that questions related to the accuracy or integrity of any part of the work are appropriately investigated and resolved. NN, former Kholodina, had a contribution to the acquisition, analysis, and interpretation of data. She was drafting the work and revising it critically for important intellectual content.

## Conflict of Interest Statement

The authors declare that the research was conducted in the absence of any commercial or financial relationships that could be construed as a potential conflict of interest.
